# Bacterial Epidemiology and Antimicrobial Resistance Profiles in Children Reported by the ISPED Program in China, 2016 to 2020

**DOI:** 10.1128/Spectrum.00283-21

**Published:** 2021-11-03

**Authors:** Pan Fu, Hongmei Xu, Chunmei Jing, Jikui Deng, Hongmei Wang, Chunzhen Hua, Yinghu Chen, Xuejun Chen, Ting Zhang, Hong Zhang, Yiping Chen, Jinhong Yang, Aiwei Lin, Shifu Wang, Qing Cao, Xing Wang, Huiling Deng, Sancheng Cao, Jianhua Hao, Wei Gao, Yuanyuan Huang, Hui Yu, Chuanqing Wang

**Affiliations:** a Department of Clinical Microbiology Laboratory, Children’s Hospital of Fudan University, National Children's Medical Center, Shanghai, China; b Nosocomial Infection Control Department, Children’s Hospital of Fudan University, National Children's Medical Center, Shanghai, China; c Infectious Disease Department, Children’s Hospital of Chonqing Medical University, Chongqing, China; d Department of Medical Laboratory, Children’s Hospital of Chonqing Medical University, Chongqing, China; e Infectious Disease Department, Shenzhen Children’s Hospital, Shenzhen, China; f Infectious Disease Department, Children’s Hospital of Zhejiang University, Zhejiang, China; g Department of Medical Laboratory, Children’s Hospital of Zhejiang University, Zhenjiang, China; h Digestive and Infectious Disease Department, Children’s Hospital of Shanghai Jiaotong University, Shanghai, China; i Department of Medical Laboratory, Children’s Hospital of Shanghai Jiaotong University, Shanghai, China; j Pediatric Infectious Disease Department, The Second Affiliated Hospital and Yuying Children’s Hospital of Wenzhou Medical University, Wenzhou, China; k Department of Medical Laboratory, The Second Affiliated Hospital and Yuying Children’s Hospital of Wenzhou Medical University, Wenzhou, China; l Infectious Disease Department, Qilu Children’s Hospital of Shandong University, Shandong, China; m Department of Medical Laboratory, Qilu Children’s Hospital of Shandong University, Shandong, China; n Infectious Disease Department, Shanghai Children’s Medical Center, Shanghai, China; o Department of Medical Laboratory, Shanghai Children’s Medical Center, Shanghai, China; p Infectious Disease Department, Xi’an Children’s Hospital, Xi’an, China; q Department of Medical Laboratory, Xi’an Children’s Hospital, Xi’an, China; r Infectious Disease Department, Children’s Hospital of Kaifeng City, Kaifeng, China; s Department of Medical Laboratory, Children’s Hospital of Kaifeng City, Kaifeng, China; t Pediatric Department, First Hospital Affiliated to Jilin University, Changchun, China; u Infectious Disease Department, Children’s Hospital of Fudan University, National Children's Medical Center, Shanghai, China; Children's Hospital Los Angeles, University of Southern California

**Keywords:** bacteria, antimicrobial resistance, children, Infectious Disease Surveillance of Pediatrics (ISPED), multidrug-resistant organisms

## Abstract

The Infectious Disease Surveillance of Pediatrics (ISPED) program was established in 2015 to monitor and analyze the trends of bacterial epidemiology and antimicrobial resistance (AMR) in children. Clinical bacterial isolates were collected from 11 tertiary care children’s hospitals in China in 2016 to 2020. Antimicrobial susceptibility testing was carried out using the Kirby-Bauer method or automated systems, with interpretation according to the Clinical and Laboratory Standards Institute 2019 breakpoints. A total of 288,377 isolates were collected, and the top 10 predominant bacteria were Escherichia coli, Streptococcus pneumoniae, Staphylococcus aureus, Haemophilus influenzae, Klebsiella pneumoniae, Moraxella catarrhalis, Streptococcus pyogenes, Staphylococcus epidermidis, Pseudomonas aeruginosa, and Acinetobacter baumannii. In 2020, the coronavirus disease 2019 (COVID-19) pandemic year, we observed a significant reduction in the proportion of respiratory tract samples (from 56.9% to 44.0%). A comparable reduction was also seen in the primary bacteria mainly isolated from respiratory tract samples, including S. pneumoniae, H. influenzae, and S. pyogenes. Multidrug-resistant organisms (MDROs) in children were commonly observed and presented higher rates of drug resistance than sensitive strains. The proportions of carbapenem-resistant K. pneumoniae (CRKP), carbapenem-resistant A. baumannii (CRAB), carbapenem-resistant P. aeruginosa (CRPA), and methicillin-resistant S. aureus (MRSA) strains were 19.7%, 46.4%%, 12.8%, and 35.0%, respectively. The proportions of CRKP, CRAB, and CRPA strains all showed decreasing trends between 2015 and 2020. Carbapenem-resistant *Enterobacteriaceae* (CRE) and CRPA gradually decreased with age, while CRAB showed the opposite trend with age. Both CRE and CRPA pose potential threats to neonates. MDROs show very high levels of AMR and have become an urgent threat to children, suggesting that effective monitoring of AMR and antimicrobial stewardship among children in China are required.

**IMPORTANCE** AMR, especially that involving multidrug-resistant organisms (MDROs), is recognized as a global threat to human health; AMR renders infections increasingly difficult to treat, constituting an enormous economic burden and producing tremendous negative impacts on patient morbidity and mortality rates. There are many surveillance programs in the world to address AMR profiles and MDRO prevalence in humans. However, published studies evaluating the overall AMR rates or MDRO distributions in children are very limited or are of mixed quality. In this study, we showed the bacterial epidemiology and resistance profiles of primary pathogens in Chinese children from 2016 to 2020 for the first time, analyzed MDRO distributions with time and with age, and described MDROs’ potential threats to children, especially low-immunity neonates. Our study will be very useful to guide antiinfection therapy in Chinese children, as well as worldwide pediatric patients.

## INTRODUCTION

Antimicrobial resistance (AMR) is most commonly associated with both high mortality rates and large medical cost burdens in health care and is recognized as one of the most serious global threats to human health (1). The gradual emergence of AMR has threatened the effective prevention and treatment of an ever-increasing range of bacterial infections. There are many AMR mechanisms in bacteria, such as reduction of drug permeability, biofilm formation, which can decrease the susceptibility to antibiotic activity, and active efflux pumps (2). Meanwhile, a growing number of novel AMR mechanisms are emerging and spreading globally (3, 4). In particular, the rapid rise in multidrug resistant organisms (MDROs) is rendering infections increasingly difficult to treat (5). MDROs included carbapenem-resistant *Enterobacteriaceae* (CRE), carbapenem-resistant Pseudomonas aeruginosa (CRPA), carbapenem-resistant Acinetobacter baumannii (CRAB), methicillin-resistant Staphylococcus aureus (MRSA), vancomycin-resistant Staphylococcus aureus (VRSA), and vancomycin-resistant enterococci (VRE) (6). Options for treating patients with MDRO infections are often extremely limited. In addition to extended hospital stays and increased morbidity and mortality rates, MDROs infections add considerable costs to the local health care system. For example, according to CDC’s antibiotic resistance threats report in the United States, more than 2.8 million MDRO infections occurred annually, causing at least 35,000 deaths and $20 billion in health care expenditures (7).

Therefore, national surveillance programs are urgently required. To date, there are several international surveillance networks, such as the Global Antimicrobial Resistance and Use Surveillance System (GLASS), the European Antimicrobial Resistance Surveillance Network (EARS-Net), the China Antimicrobial Resistance Surveillance System (CARSS), and the China Antimicrobial Surveillance Network (CHINET) (8–10). However, these networks mainly focus on AMR surveillance in adult populations, instead of addressing that in children. Additionally, the prevalence distributions and AMR patterns of bacteria isolated from children are quite unlike those from adults, because children are not just “little adults” in the AMR era (11–13). Taking Streptococcus pneumoniae as an example, the rate of S. pneumoniae carriage is high in children but low in adults (53% versus 4%), and S. pneumoniae serotype distribution and antibiotic resistance patterns are also very different between adults and children (14, 15). Therefore, an AMR surveillance network unique to children is essential for pediatric antimicrobial stewardship. To focus on AMR surveillance in pediatric patients, we established the Infectious Disease Surveillance of Pediatrics (ISPED) program in 2015, which currently includes 11 tertiary care children’s hospitals covering nine provinces and autonomous regions of mainland China.

Here, we present a large group of data on bacteria in the past 5 years from ISPED, as well as a comprehensive analysis of the evolution of bacterial epidemiology and the AMR profiles. The current prevalence of MDROs in Chinese children was also investigated in the study.

## RESULTS

### Distribution of clinical isolates.

From 2016 to 2020, a total of 288,377 bacterial strains isolated from children were enrolled in the ISPED program, of which 12.8% (37,050 strains) were collected from outpatients and 87.2% (251,327) were collected from inpatients. There were 121,265 Gram-positive bacteria (42.1%) and 167,112 Gram-negative bacteria (57.9%). The top 10 bacteria were Escherichia coli (13.4%), S. pneumoniae (11.8%), S. aureus (11.0%), Haemophilus influenzae (10.1%), Klebsiella pneumoniae (6.7%), Moraxella catarrhalis (6.3%), Streptococcus pyogenes (4.7%), Streptococcus epidermidis (4.4%), P. aeruginosa (3.6%), and A. baumannii (3.1%). Most of the strains came from the respiratory tract (53.9%), followed by blood (10.4%) and urine (10.0%). Notably, the constituent proportion in the respiratory tract dropped from 56.9% in 2016 to 44.0% in 2020 (see Table S1 in the supplemental material).

S. pneumoniae, H. influenzae, and M. catarrhalis were the main bacteria isolated from lower respiratory tract samples, the proportions of which were 22.5%, 18.8%, and 12.5%, respectively. S. pyogenes was the dominant species in upper respiratory tract samples (75.4%). E. coli was the primary species isolated from urine and abscess samples, and the proportions were 39.7% and 38.3%, respectively. S. aureus was the primary species isolated from wound samples (40.4%). S. epidermidis, Staphylococcus hominis, and E. coli were mainly isolated from patients’ blood samples (Table 1). However, coagulase-negative Staphylococcus (CoNS) strains in blood cultures were mostly regarded as contamination (16, 17). Making a correct diagnosis of pathogenicity (versus contamination) was challenging because we lacked CoNS strain characteristics and patients’ clinic information, such as the diagnosis or laboratory testing results.

**TABLE 1:** Distributions of the top five pathogens from different specimens reported by the ISPED program in 2016 to 2020

Specimen type	Species (%)				
	Pathogen 1	Pathogen 2	Pathogen 3	Pathogen 4	Pathogen 5
Lower respiratory tract	S. pneumoniae (22.5)	H. influenzae (18.8)	M. catarrhalis (12.5)	S. aureus (12.4)	K. pneumoniae (7.8)
Upper respiratory tract	S. pyogenes (75.4)	S. aureus (9.6)	H. influenzae (4.8)	S. pneumoniae (4.6)	K. pneumoniae (1.7)
Blood	S. epidermidis (27.9)	S. hominis (18.4)	E. coli (6.0)	Stenotrophomonas maltophilia (5.4)	K. pneumoniae (4.5)
Urine	E. coli (39.7)	E. faecium (13.3)	K. pneumoniae (10.5)	E. faecalis (9.8)	Proteus mirabilis (4.1)
Wound	S. aureus (40.7)	E. coli (16.0)	P. aeruginosa (8.2)	A. baumannii (3.9)	K. pneumoniae (3.9)
Abscess	E. coli (38.3)	S. aureus (30.5)	P. aeruginosa (9.2)	K. pneumoniae (4.1)	Enterococcus avium (2.0)

**FIG 1:**
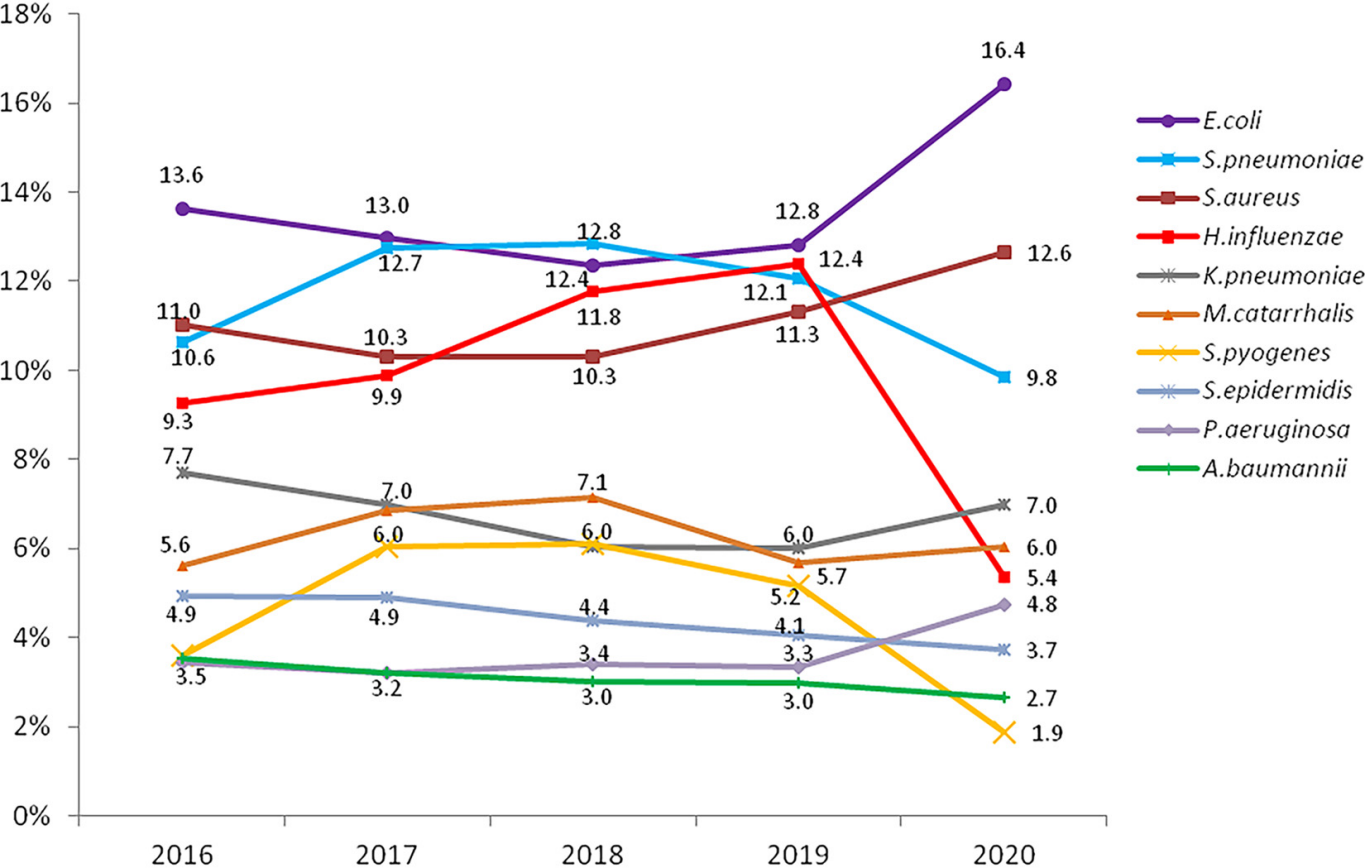
Distribution trends of main pathogens reported by the ISPED program in 2016 to 2020.

The bacterial spectrum has changed in the past 5 years, especially in 2020, the coronavirus disease 2019 (COVID-19) pandemic year. The constituent proportions of H. influenzae, S. pneumoniae, and S. pyogenes, which were mainly isolated from respiratory tract samples, decreased sharply in 2020. The proportions of S. pneumoniae and S. pyogenes decreased from the maximum values of 12.8% and 6.0%, respectively, to 9.8% and 1.9% in 2020. Notably, the proportion of H. influenzae, after showing a gradually increasing trend from 2016 (9.3%) to 2019 (12.4%), decreased greatly in 2020 (5.4%) (Fig. 1).

### Distribution of MDROs.

**FIG 2:**
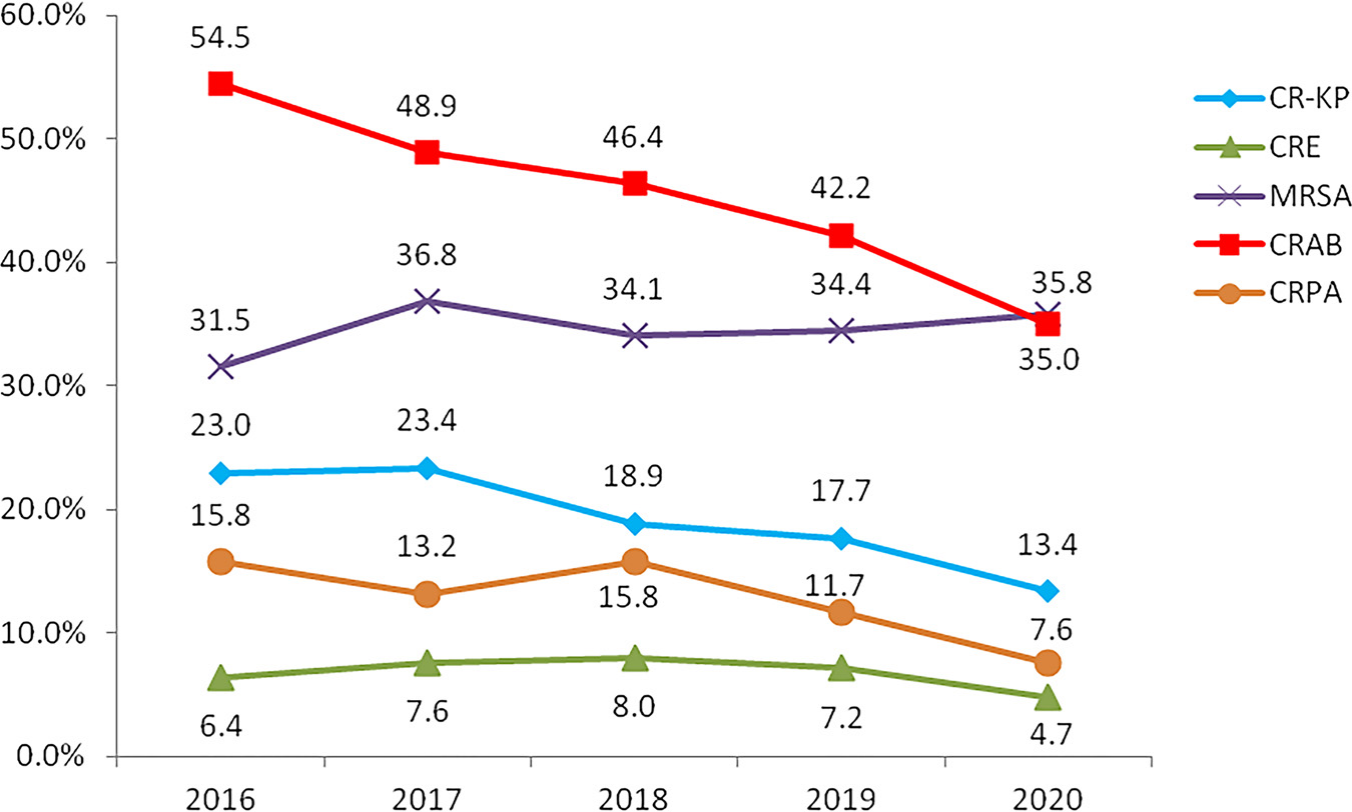
Distribution trends of MDROs reported by the ISPED program in 2016 to 2020.

The proportions of MRSA strains ranged from 31.5% to 36.8% in the past 5 years. We obtained 6.8% CRE strains (5,726 strains) among *Enterobacteriaceae* strains. Among those CRE strains, 54.1% were carbapenem-resistant K. pneumoniae (CRKP). By analyzing the changing trends for CRKP, CRAB, and CRPA, we found that all of them showed gradually decreasing trends from 2015 to 2020, from the maximum values of 23.4%, 54.5%, and 15.8%, respectively, to 13.4%, 35.0%, and 7.6% in 2020 (Fig. 2). The average proportions of MRSA, CRKP, CRAB, and CRPA were 34.5%, 19.3%, 45.4%, and 12.8%, respectively.

**FIG 3:**
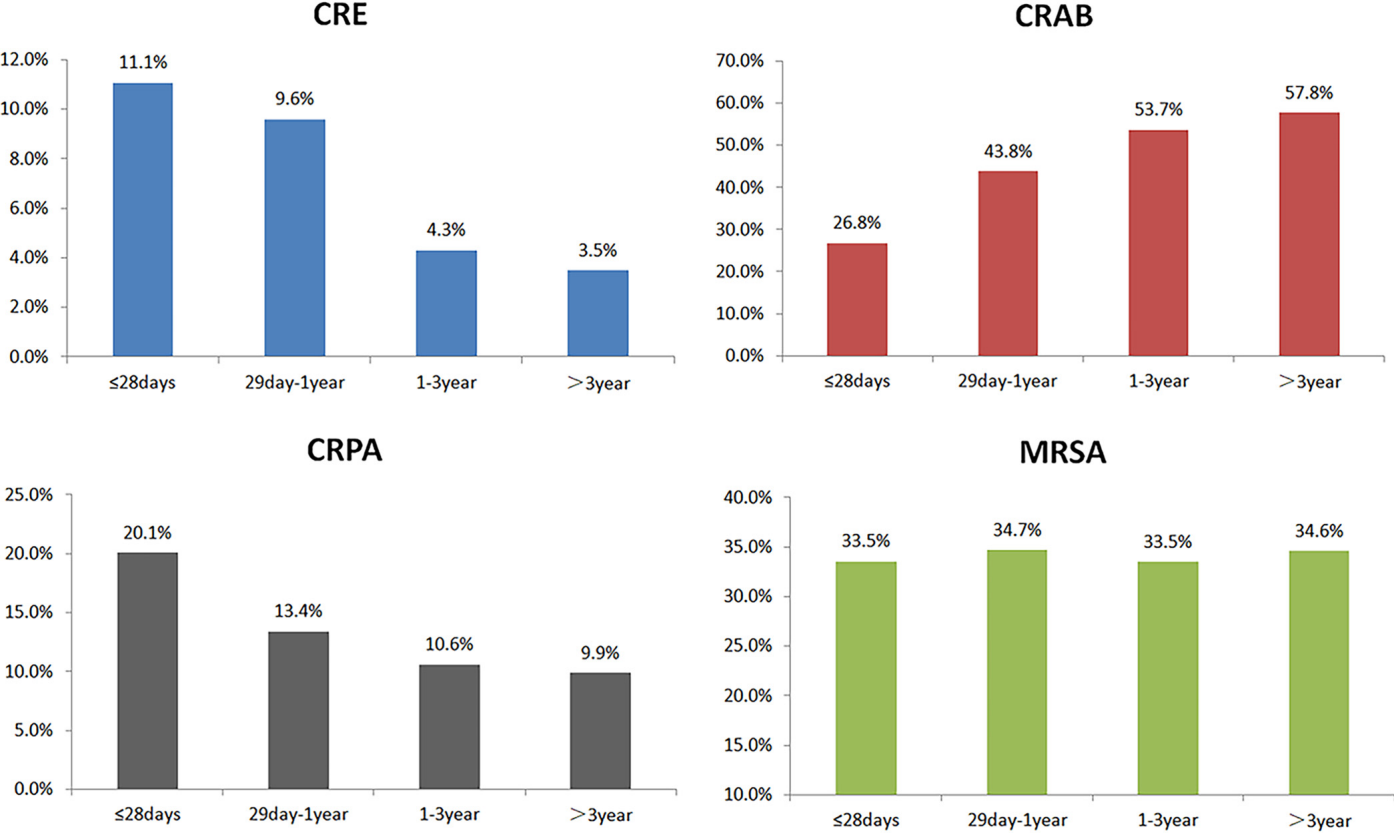
Distribution trends of MDROs in different age groups, as reported by the ISPED program.

We compared the MDRO proportions in the neonatal group and the nonneonatal group. Interestingly, CRE and CRPA proportions were greater in the neonatal group (11.1% and 20.1%, respectively) than in the nonneonatal group (5.5% and 12.0%, respectively; *P* < 0.05). However, the proportion of CRAB, which accounted for 26.8% in neonates, was much smaller in nonneonates (53.4%; *P* < 0.05) (Table 2). After further dividing MDROs into broader age groups, we found that the distributions of CRE, CRPA, and CRAB strains varied among different age groups. The proportions of CRE and CRPA gradually decreased with age, from 11.1% to 3.5% and from 20.1% to 9.9%, respectively, while the proportion of CRAB showed an opposite trend, increasing from 26.8% to 57.8% with age (Fig. 3).

**TABLE 2:** Distributions of MDROs among neonates and nonneonates in 2016 to 2020

MDRO and group	Proportion (%) in ISPED program in:						*P*
	2016	2017	2018	2019	2020	Total	
CRE							
Neonates	10.2	14.1	14.1	10.3	5.5	11.1	0.01
Nonneonates	5.1	5.4	6.1	6.2	4.5	5.5	
CRKP							
Neonates	19.1	24.8	22.2	13.2	10.3	18.7	0.54
Nonneonates	25.4	22.5	17.0	20.3	14.8	20.3	
CRAB							
Neonates	35.3	25.5	29.5	19.6	16.2	26.8	<0.001
Nonneonates	62.5	58.7	51.9	49.3	39.7	53.4	
CRPA							
Neonates	19.8	22.7	23.4	16.5	13.6	20.1	0.016
Nonneonates	15.2	11.6	14.9	11.3	7.2	12.0	
MRSA							
Neonates	29.2	34.2	34.1	34.1	36.1	33.5	0.34
Nonneonates	32.6	38.2	34.1	34.6	35.7	35.0	

The distributions of MDROs in inpatients and outpatients were also very distinct. CRE, CRKP, and CRAB isolates were detected much more commonly from inpatients than from outpatients, but MRSA was more prevalent among outpatients than among inpatients (*P* < 0.05) (Table 3).

**TABLE 3:** Distributions of MDROs in inpatients and outpatients in 2016 to 2020

MDRO and group	Proportion (%) in ISPED program in:						*P*
	2016	2017	2018	2019	2020	Total	
CRE							
Inpatients	9.4	9.8	9.5	9.4	6.3	8.8	<0.001
Outpatients	2.3	2.2	3.9	4.4	1.1	3.0	
CRKP							
Inpatients	23.9	24.7	19.5	18.1	13.9	19.8	0.005
Outpatients	9.3	10.3	12.3	13.5	3.4	10.7	
CRAB							
Inpatients	55.5	49.7	46.7	42.2	35.5	45.5	0.017
Outpatients	12.5	24.4	36.8	41.2	18.2	30.9	
CRPA							
Inpatients	16.1	13.4	16.5	10.3	6.2	11.6	0.09
Outpatients	6.7	10.9	10.3	8.5	2.9	8.1	
MRSA							
Inpatients	31.8	37.2	34.8	35.0	36.5	28.9	0.001
Outpatients	29.4	32.1	27.7	27.7	27.4	35.0	

### AMR trends in the main bacteria.

**(i) *Staphylococcus*.** For S. aureus, the rate of resistance to penicillin (92.3% to 93.2%) was the highest. About 35% of S. aureus strains were resistant to oxacillin (see Table S2). The MRSA strains exhibited significantly higher rates of resistance to erythromycin and clindamycin, compared with methicillin-susceptible S. aureus (MSSA) strains (Table 4).

**TABLE 4:** AMR rates of MRSA and MSSA strains reported by the ISPED program in 2016 to 2020

Antibiotic	Rate of resistance (%) for:	
	MRSA (*n* = 11,128)	MSSA (*n* = 20,667)
Penicillin G	100	89
Oxacillin	100	0
Gentamicin	3.7	7.6
Rifampin	1.6	0.5
Ciprofloxacin	7.5	4.4
Levofloxacin	5.7	4.1
Moxifloxacin	4.3	3.2
Trimethoprim-sulfamethoxazole	5.2	11.6
Clindamycin	62.8	28.5
Erythromycin	78.2	51.9
Linezolid	0	0
Vancomycin	0	0

A total of 23,670 CoNS strains were enrolled. The rate of resistance to penicillin among them was up to 95.4%. More than 80% of those strains were resistant to oxacillin and erythromycin (see Table S2).

**(ii) *Streptococcus*.** The rates of resistance to clindamycin and erythromycin were very high (>90%) for both S. pneumoniae and S. pyogenes. The majority of S. pneumoniae strains (99.1%) were collected from non-cerebrospinal fluid samples. All of the S. pyogenes strains were sensitive to penicillin, while penicillin-nonsusceptible S. pneumoniae (PNSP) strain rates ranged from 9.6% to 20.7% in 2016 to 2020 (see Table S3).

**(iii) *Enterococcus*.** We collected 4,394 Enterococcus faecalis strains and 6,799 Enterococcus faecium strains from 2016 to 2020. The E. faecium strains showed higher rates of resistance to ampicillin, ciprofloxacin, levofloxacin, and erythromycin, while the E. faecalis strains were more sensitive to most tested antimicrobials (<15%), except for high gentamicin (21.3% to 33.7%) and erythromycin (57.2% to 64.3%) (see Table S4).

**(iv) *Enterobacteriaceae*.** CRE strains accounted for 6.8% among *Enterobacteriaceae* strains, which were more resistant to most detected antimicrobials than carbapenem-susceptible *Enterobacteriaceae* (CSE) strains (Table 5). The AMR profiles of most antimicrobials in E. coli showed no obvious changes from 2016 to 2020. Rates of resistance to ampicillin-sulbactam were much higher, ranging from 47.8% to 61.1%. More than 30% of strains were resistant to ciprofloxacin, aztreonam, and levofloxacin. The rates of resistance to meropenem and amikacin were very low, ranging from 2.2% to 3.7% and from 0.9% to 1.3%, respectively.

**TABLE 5:** AMR rates of carbapenem-resistant and carbapenem-susceptible strains reported by the ISPED program in 2016 to 2020

Antibiotic	Rate of resistance (%) for^*a*^:							
	Enterobacter		K. pneumoniae		A. baumannii		P. aeruginosa	
	CRE (*n* = 5,726)	CSE (*n* = 78,131)	CRKP (*n* = 3,822)	CSKP (*n* = 11,545)	CRAB (*n* = 4,157)	CSAB (*n* = 4,793)	CRPA (*n* = 1,315)	CSPA (*n* = 8,944)
Ampicillin	97.7	80.5	NA	NA	99.9	27.6	NA	NA
Piperacillin	91.1	63.9	99.5	60.6	99	17.1	41	3.5
Ampicillin-sulbactam	95.5	43.3	99	47.6	90.5	2.8	98.8	99
Piperacillin-tazobactam	79.8	2.6	90.4	5.5	96.5	1.9	29.7	1.7
Cefuroxime	97	48.3	99.9	47.9	100	87.3	100	99.7
Ceftazidime	92.4	20.7	97.9	27.2	95.4	2.5	35.7	2.8
Cefepime	85	16.7	92	23.4	96.5	2.2	35.2	1.5
Aztreonam	78.4	28.6	84.2	32.8	96.7	39.3	47.9	6
Amikacin	17.2	0.9	26.8	1.1	70.6	1	18.7	0.4
Gentamicin	38.8	23.7	44.8	16.4	87.1	2.6	20.1	1.7
Ciprofloxacin	54.9	28.4	50.6	25.3	89.6	1.7	21.9	2.2
Levofloxacin	37.4	18.3	32.9	7.6	60.1	0.8	23.3	2.5
Trimethoprim-sulfamethoxazole	45.8	40.5	40.2	34.5	76.2	4.9	91.8	90.3

a NA, not available.

**FIG 4:**
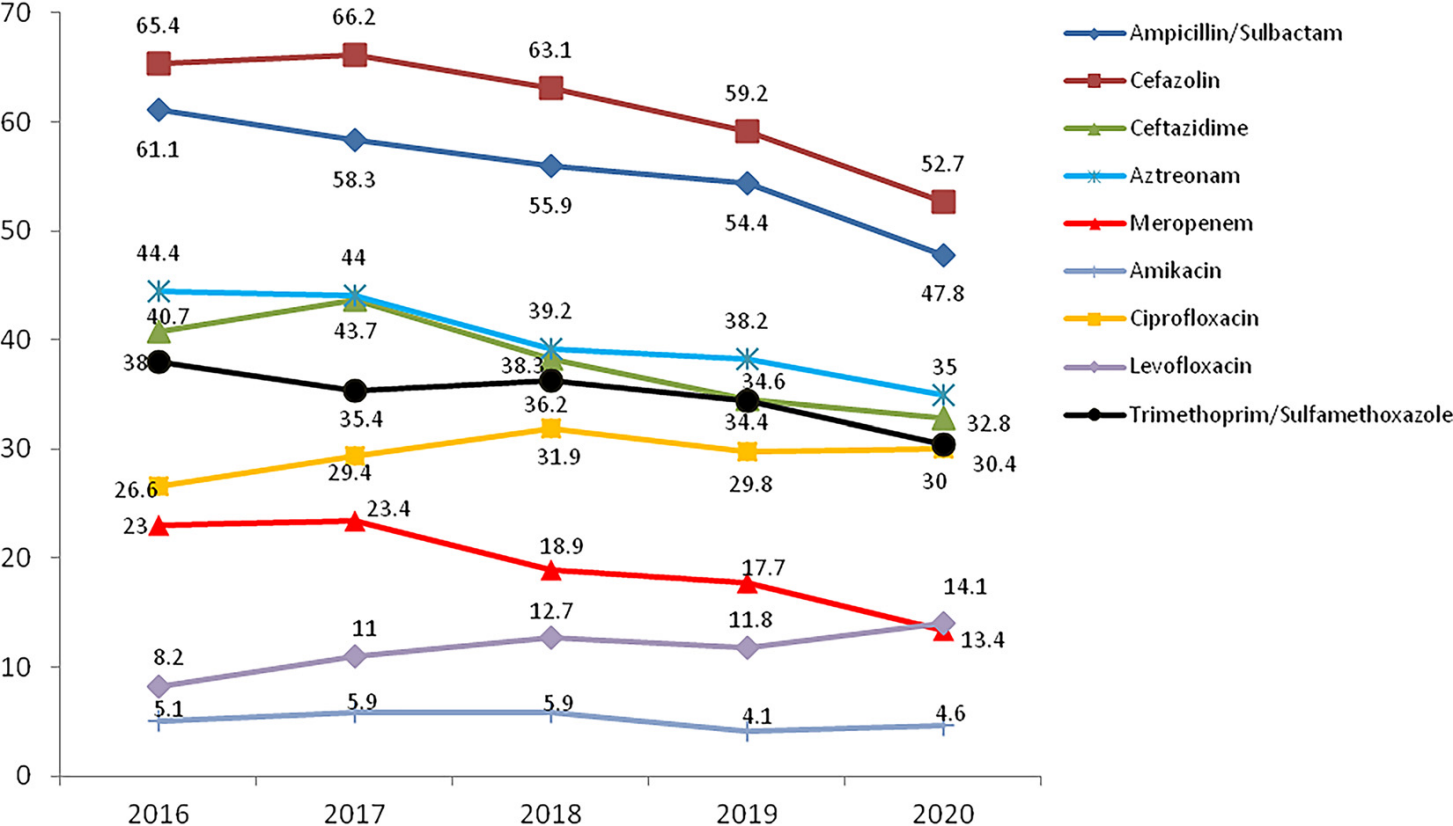
Resistance profile (%) of K. pneumoniae for nine main antimicrobials, as reported by the ISPED program in 2016 to 2020.

K. pneumoniae showed high rates of resistance to cefazolin and ampicillin-sulbactam. Most antimicrobials exhibited decreasing trends from 2016 to 2020. Notably, the proportion of meropenem-resistant K. pneumoniae decreased gradually from 23% in 2016 to 13.4% in 2020 (Fig. 4). Carbapenem-susceptible K. pneumoniae (CSKP) strains were much more sensitive to most tested antimicrobials than CRKP isolates (Table 5).

### Nonfermentive Gram-negative bacilli.

**FIG 5:**
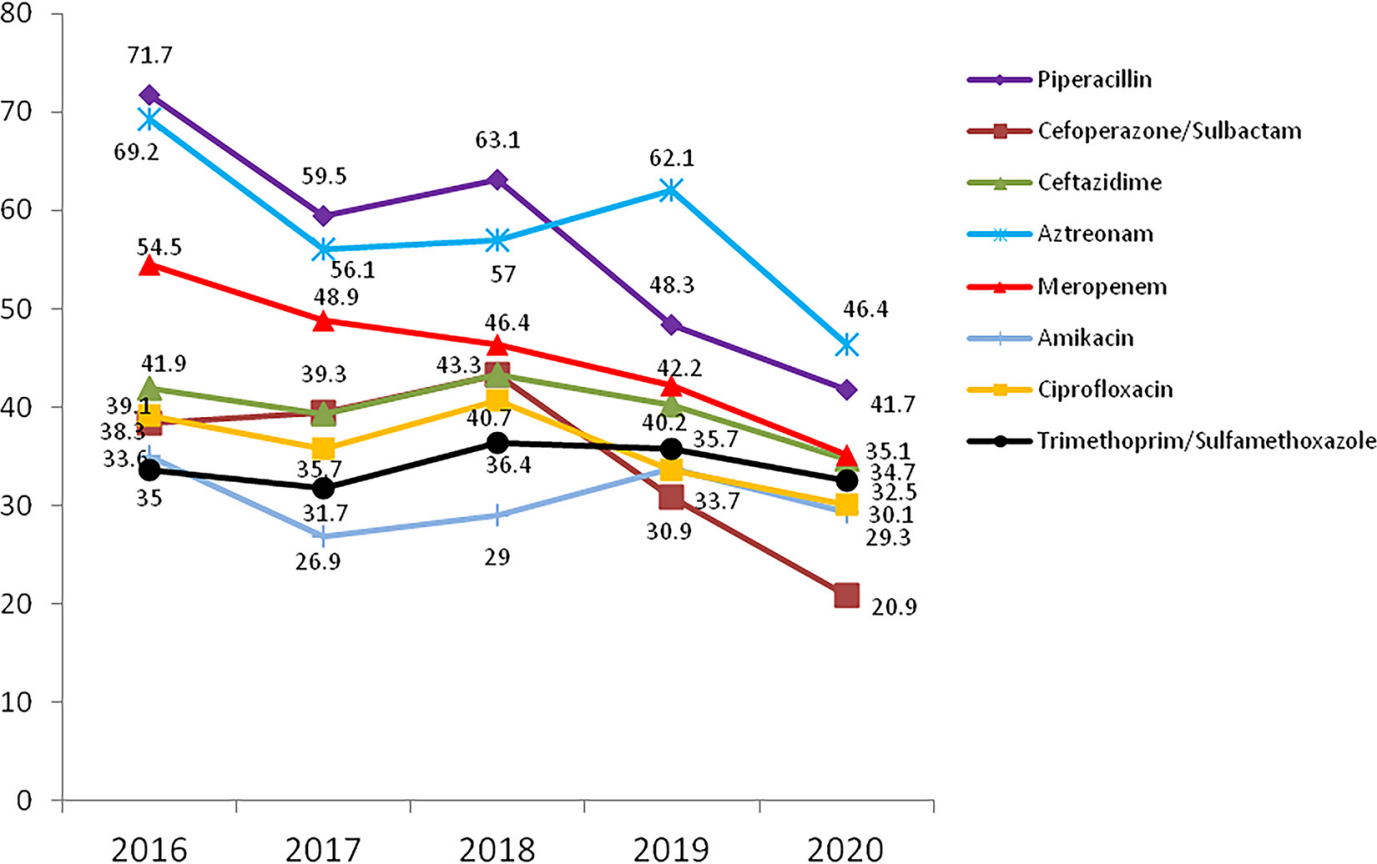
Resistance profile (%) of A. baumannii for eight main antimicrobials, as reported by the ISPED program in 2016 to 2020.

A. baumannii showed moderate or high rates of resistance to most tested antimicrobials. Resistance to piperacillin, cefoperazone-sulbactam, and aztreonam displayed decreasing trends from 2016 to 2020. The proportions of meropenem-resistant A. baumannii strains also decreased gradually from 54.5% in 2016 to 34.7% in 2020 (Fig. 5).

**FIG 6:**
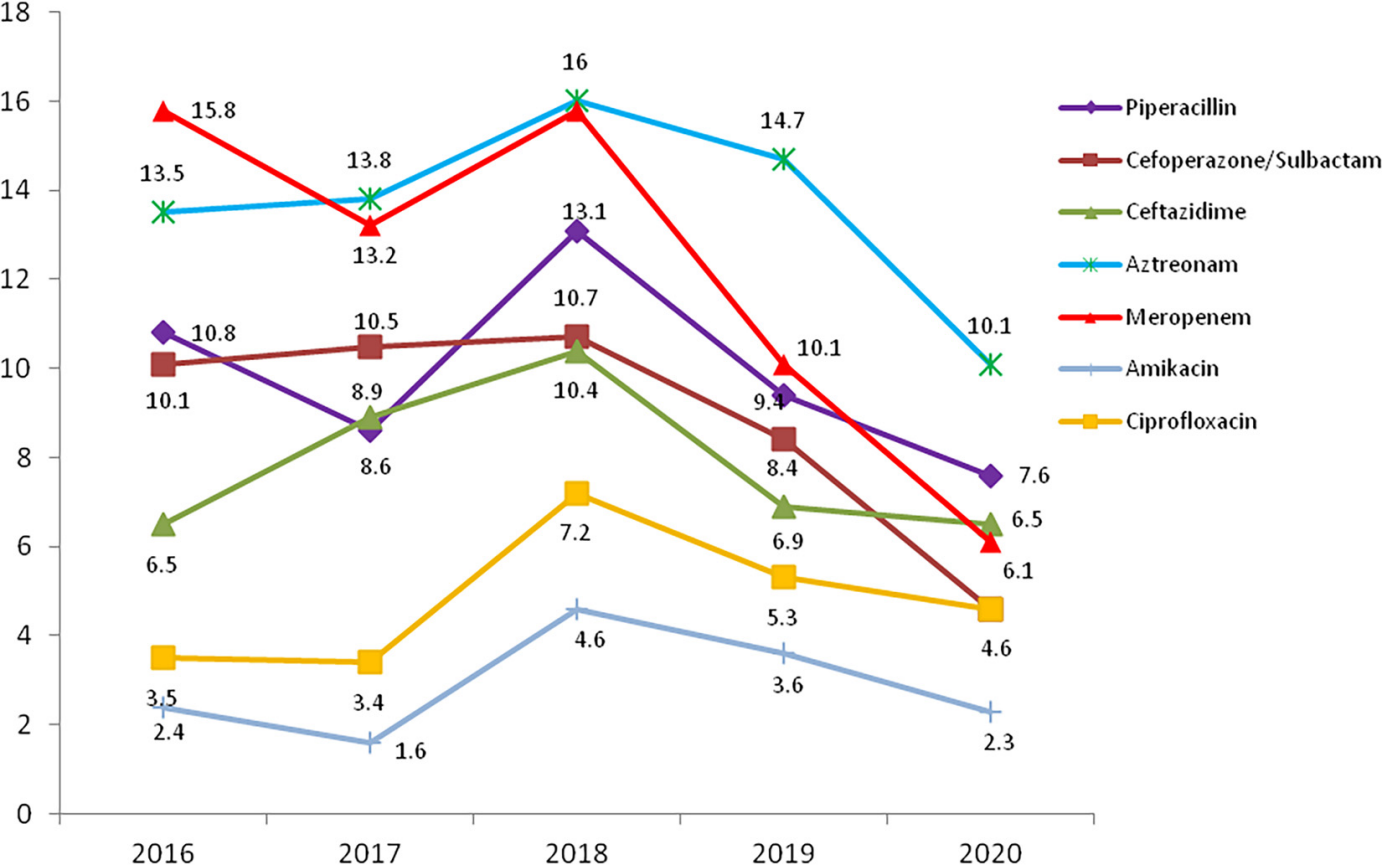
Resistance profile (%) of P. aeruginosa for seven main antimicrobials, as reported by the ISPED program in 2016 to 2020.

AMR in P. aeruginosa was much milder than that in A. baumannii. The proportions of meropenem-resistant P. aeruginosa showed decreasing but fluctuating trends, from 15.8% in 2016 to 6.1% in 2020 (Fig. 6). Both CRAB and CRPA strains showed much higher rates of resistance to most tested antimicrobials, compared with carbapenem-susceptible A. baumannii (CSAB) and carbapenem-susceptible P. aeruginosa (CSPA), as shown in Table 5.

### Fastidious bacteria.

H. influenzae and M. catarrhalis were listed in the top 10 primary bacteria in children. More than 95% of M. catarrhalis strains and 60% of H. influenzae strains produced β-lactamases. M. catarrhalis was more sensitive to most antibiotics (see Table S5).

### Salmonella.

A total of 8,498 Salmonella strains, which were mainly collected from stool samples (94.0%), were enrolled in this study. Salmonella isolates presented high rates of resistance to ampicillin (>75%) and moderate rates of resistant to ampicillin-sulbactam, trimethoprim-sulfamethoxazole, and chloramphenicol (see Table S6). Most of the Salmonella strains (98.9%) were nontyphoidal Salmonella; the proportion of Typhi/Paratyphi Salmonella was 1.1% (94/8,498 strains).

## DISCUSSION

AMR is an established global threat and carries an increased risk of infection-associated death for children infected with MDROs (18). Long-term global and national surveillance of AMR trends is thus required to guide clinical antiinfection treatment. To our knowledge, we are the first to describe and analyze the overall AMR profiles of common bacteria isolated from children in China, indicating that MDROs were commonly detected among children and presented much greater AMR, compared with the sensitive strains. From 2016 to 2020, a total of 288,377 bacterial strains were enrolled in the ISPED program; the top 10 predominant bacteria isolated from children were E. coli, S. pneumoniae, S. aureus, H. influenzae, K. pneumoniae, M. catarrhalis, S. pyogenes, S. epidermidis, P. aeruginosa, and A. baumannii, which was largely different from the international reports from CARSS and EARS-Net (8, 9).

Pulmonary infections remain a major cause of death for infants and children worldwide and are responsible for a substantial burden of morbidity (19). In our study, more than one-half of the bacterial strains were collected from respiratory tract samples, indicating that pediatric bacterial respiratory infections are very common in China. In 2020, however, the COVID-19 pandemic year, we observed a significant reduction in the constituent proportion of respiratory tract samples (from 56.9% to 44.0%). A comparable reduction was also seen for the primary bacteria that were mainly isolated from respiratory tract samples, including S. pneumoniae, H. influenzae and S. pyogenes. Therefore, we considered that the control measures during COVID-19 lockdown, such as keeping social distance and wearing masks, blocked or slowed the spread of the microorganisms, especially the bacteria that mainly caused respiratory infections. These findings were consistent with the large reductions in respiratory infection cases during the COVID-19 pandemic-related lockdown period that were reported previously (20–23).

MDROs constitute an enormous economic burden, given their tremendous negative impact on patient morbidity and mortality rates (24). In the 2019 U.S. Centers for Disease Control and Prevention Antibiotic Resistance Report, CRE remained in the most urgent AMR threat category, along with CRAB, CRPA, and other MDROs (25). Published studies evaluating MDROs among pediatric patients are very limited or are of mixed quality. Surveillance of MDROs is a critically important component of the ISPED program. In our study, MRDOs were commonly detected among children in China. The proportions of CRAB, CRKP, and CRPA strains in our study were 46.4%, 19.3%, and 12.8%, respectively, much lower than the proportions for adults in the CHINET report (78.1%, 26.3%, and 30.7%, respectively) (26).

MDRO distributions were very distinct with time and with age. CRKP, CRPA, and CRAB proportions presented noticeable decreasing trends from 2016 to 2020, which were very different and even showed opposite trends, compared with those for adult patients (26). Decreased MDRO proportions among children may be directly related to good implementation of infection control measures and antimicrobial stewardship in pediatric clinic practice. Moreover, AMR genes prevalent among children were also distinct from those prevalent among adults; therefore, their transfer mechanisms might be varied, showing different spread trends or patterns. For example, *bla*_NDM_ was the primary carbapenem resistance gene in CRKP among children, while *bla*_KPC_ was dominant among adults (27, 28). MDRO distributions were closely related to age. In our study, CRE and CRPA proportions gradually decreased with age, while the proportion of CRAB showed an increasing trend with age. By comparing the MDROs in the neonatal group and the nonneonatal group, we noticed a marked difference between the two groups. The rates of CRE and CRPA were much higher in the neonatal group than in the nonneonatal group. The high level of MDROs in the neonatal group was possibly related to the immature immunity of neonates (29) and maternal exposure, especially exposure to *Enterobacteriaceae* during labor. The mortality rate for neonates with CRE sepsis was reported to be up to 33.3% (30). Neonates have lower immunity and are prone to bacterial infections; therefore, it is urgent to intensify antimicrobial stewardship efforts and to address infection control and prevention of MDROs in neonatal units.

MDROs can be easily transferred among patients. Previous research showed that MDRO transfer events were observed in 18.5% of patient encounters and occurred early in the admissions (31). In this study, CRE, CRPA, and CRAB were more prevalent among inpatients than among outpatients, revealing that hospital stays and patient contacts were the risk factors for MDRO infections. MRSA accounted for 31.5% to 36.8% of S. aureus strains, exhibiting much higher rates of resistance to erythromycin and clindamycin, compared with MSSA strains. Meanwhile, CRE, CRKP, CRPA, and CRAB strains all showed notably higher rates of resistance to most tested antimicrobials, compared with carbapenem-susceptible isolates, which indicated the difficulty of empirical antimicrobial therapy in children infected by MDROs.

In conclusion, the bacterial epidemiology and resistance in children are characteristic and quite different from those for the adult population. MDROs presented much higher AMR profiles and have become an urgent threat to children, with CRKP, CRAB, and CRPA strains showing decreasing but fluctuating trends between 2015 and 2020. Keeping effective and continuous surveillance on the trends of bacterial epidemiology and AMR profiles among children is of great significance in China.

## MATERIALS AND METHODS

### Enrollment of bacteria and patients.

From 2016 to 2020, all unduplicated aerobic bacterial strains were collected from 11 tertiary care children’s hospital in China. These hospitals represented nine provinces or autonomous cities (Guangdong province, Jiangsu province, Zhejiang province, Shandong province, Shanxi province, Henan province, Jilin province, Shanghai city, and Chongqing city) across the mainland of China. For repeated strains, only the first isolate from the same species and the same patient was enrolled in this study. Species identification was performed by standard biochemical methods. Neonatal patients were defined as children with ages at discharge between 0 and 28 days (inclusive), while nonneonatal patients were defined as children with ages of 28 days to 18 years (exclusive).

### Isolate inclusion and exclusion criteria.

Unified standards were used stringently in each hospital. Inclusion and exclusion criteria for different samples were as follows; upper respiratory tract, only β-hemolytic Streptococcus was included; lower respiratory tract, most opportunistic pathogens, such as α-hemolytic Streptococcus and *Neisseria* spp., were excluded; blood, all unduplicated aerobic bacteria collected from blood cultures were included; stool, only enteropathogenic bacteria were included; urine, only samples with bacterial levels of >10^4^ CFU/ml and ≤2 bacterial species were included.

### Antimicrobial susceptibility.

Antimicrobial susceptibility tests were carried out using the Kirby-Bauer method or automated systems, with interpretation according to the Clinical and Laboratory Standards Institute (CLSI) 2019 breakpoints (32). The penicillin susceptibility of S. pneumoniae was detected by Etest, and the breakpoints for meningitis and nonmeningitis samples were different.

CRE strains were defined as *Enterobacteriaceae* strains that presented resistance to one of ertapenem, imipenem, or meropenem. CRAB and CRPA strains were identified as imipenem- or meropenem-resistant Acinetobacter baumannii and Pseudomonas aeruginosa, respectively. PNSP strains were defined as penicillin-intermediate or resistant strains. PNSP strains were tested by penicillin Etest, and results were interpreted according to the 2019 CLSI standard.

### β-Lactamase test.

The Oxoid Touch Stick β-lactamase product was used to detect β-lactamase activity according to the instructions. This product exhibits a rapid distinctive color change from yellow to red. A positive result was defined as a disk color change from yellow to pink/red. A negative result was defined as no color change.

### Reference strains.

Staphylococcus aureus ATCC 25923, Enterococcus faecalis ATCC 29212, Escherichia coli ATCC 25922, and P. aeruginosa ATCC 27853 were included to ensure reproducibility of the antibiotic susceptibility testing procedure.

### Statistical analysis.

Statistics analyses were performed by using GraphPad Prism v7.0 (GraphPad Software, Inc., San Diego, CA, USA). Differences among the groups were analyzed with independent-sample *t* tests. Two-sided *P* values of less than 0.05 were considered statistically significant.
