# Supplementary material for: Bacterial Epidemiology and Antimicrobial Resistance Profiles in Children Reported by the ISPED Program in China, 2016 to 2020
Source: Microbiol Spectr. 2021 Nov 3;9(3):e00283-21. doi: 10.1128/Spectrum.00283-21 (PMC8567242; doi:10.1128/Spectrum.00283-21)
Supplement: SUPPLEMENTAL FILE 1 — Supplemental material. Download SPECTRUM00283-21_Supp_1_seq11.pdf, PDF file, 0.3 MB [file spectrum00283-21_supp_1_seq11.pdf]

S1. Constituent ratio of different specimens reported by ISPED program during 2016 to 2020

Specimen		Suveilance years reported by ISPED program					
		2016(n=58,628)	2017(n=59,565)	2018(n=62,465)	2019(n=64,933)	2020(n=42,786)	Total
Respiratory tract	Lower	51.5%	49.9%	49.2%	49.0%	41.3%	48.6%
	Upper	5.4%	6.9%	7.1%	5.0%	2.7%	5.3%
Blood		10.9%	10.8%	10.0%	9.8%	10.4%	10.4%
Urine		9.5%	9.5%	9.9%	9.2%	12.5%	10.0%
Pus		7.1%	7.2%	8.8%	9.9%	12.4%	8.9%

S2. AMR profiles of *S.aureus* and *CNS* reported by ISPED program during 2016 to 2020

Antibiotic name	*S.aureus*(n=31,795)					*CNS*(n=23,670)				
	2016	2017	2018	2019	2020	2016	2017	2018	2019	2020
Penicillin G	93.2	93.2	92.3	92.7	92.5	94.5	95.4	94.9	93.7	93.1
Oxacillin	31.5	36.8	34.1	34.4	35.8	80	82.4	81.9	81.6	80
Gentamicin	7.3	6.6	6.5	6.1	5.3	21	17.5	13	12.1	12.5
Rifampin	1.1	1.1	0.6	0.8	0.8	8.9	8.1	8.1	7.1	6.4
Ciprofloxacin	5.7	5.5	4.9	5.5	5.3	26.2	26	25.3	26.1	29.9
Levofloxacin	4.8	4.3	3.9	4.9	5.6	26.1	26.3	28.3	28.8	31.1
Moxifloxacin	3.6	3	3.2	3.9	4.2	11	12.9	12.6	12.8	13.6
Trimethoprim/Sulfamethoxazole	8.7	8.3	9.4	9.6	9.4	44.3	44.6	42.8	38.2	35.9
Clindamycin	36.1	44.6	38.1	39.2	44.5	34.7	41	34.4	42.9	47.8
Erythromycin	60.8	81	76.3	67.4	81.8	80.5	79.2	81.2	81	81.4
Linezolid	0	0	0	0	0	0	0	0	0	0
Vancomycin	0	0	0	0	0	0	0	0	0	0
Teicoplanin	0	0	0	0	0	0	0	0	0	0

S3. AMR profiles of *S.pneumoniae* and *S.pyogenes* reported by ISPED program during 2016 to 2020

Antibiotic name	*S.pneumoniae*(n=33,894)					*S.pyogenes*(n=13,657)				
	2016	2017	2018	2019	2020	2016	2017	2018	2019	2020
Penicillin G*	18.5	20.7	9.6	16.5	11.7	0	0	0	0	0
Levofloxacin	0.2	0.1	0.2	0.2	0.2	0.1	0.2	0.1	0.2	0.1
Trimethoprim/Sulfamethoxazole	74.6	71.3	70.3	64.8	57.8	NA	NA	NA	NA	NA
Clindamycin	95.9	94.2	93.6	96.4	95	92.4	94.5	94	91.3	92

Erythromycin	97.9	97.7	97.8	98.8	97.7	95.6	95.3	95.7	93.5	92.8
Linezolid	0	0	0	0	0	0	0	0	0	0
Vancomycin	0	0	0	0	0	0	0	0	0	0

NA. Not available

S4. AMR profiles of *E.faecalis* and *E.faecium* reported by ISPED program during 2016 to 2020

Antibiotic name	*E.faecalis* (n=4,394)					*E.faecium*(n=6,799)				
	2016	2017	2018	2019	2020	2016	2017	2018	2019	2020
Ampicillin	3.1	2.7	3.5	2.8	3.6	94	95	92.9	91.3	89.2
Gentamicin-High	21.3	33.7	28.4	23.5	31.5	26.8	46.5	41.1	36.8	38.9
Ciprofloxacin	6.6	7.2	8.3	12.3	14.7	87.2	82.7	74.5	71.7	67
Levofloxacin	6.9	7.8	9.2	10	11.7	80.3	75.2	66.4	57.4	55.1
Erythromycin	64.3	64	63.5	62.8	57.2	76.3	76.5	79.5	79.7	75.3
Nitrofurantoin	0.5	0.4	0.9	0.5	1.8	9.9	9.2	8.1	8.9	10.1
Linezolid	0	0	0	0	0	0	0	0	0	0
Vancomycin	0	0	0	0	0	0	0	0	0	0
Teicoplanin	0	0	0	0	0	0	0	0	0	0

S5. AMR profiles of *H.influenzae* and *M.catarrhalis* reported by ISPED program during 2016 to 2020

Antibiotic name	*H.influenzae*(n=29,008)					*M.catarrhalis*(n=18,085)				
	2016	2017	2018	2019	2020	2016	2017	2018	2019	2020
Beta-lactamase	67.4	59.2	60.3	63.3	64.7	96.1	97.2	98.4	99.1	98.9
Amoxicillin/Clavulanic acid	39.5	38	36.5	34	24	1.6	1.4	1.8	2.3	0.8
Ampicillin/Sulbactam	29.9	33.6	43	52.2	48.6	NA	NA	NA	NA	NA
Cefuroxime	52.3	53.5	52.7	57.8	51.9	15.7	11.3	2.9	1.8	1.2
Cefotaxime*	9.7	8.5	8.4	5.3	7.6	1.9	2	1.3	0	0
Levofloxacin*	0.6	0.2	0.3	0	0.3	0	0	1.1	0.9	0
Trimethoprim/Sulfamethoxazole	55.9	72.7	71.7	74.2	65.7	2.7	17.2	8.8	8.1	9.1
Azithromycin*	30.3	29.7	37.6	44	42.5	52.9	49.3	55.2	45.5	40.5
Chloramphenicol	6.2	5.4	4.3	4.3	4.5	2.3	0.5	0.1	0.9	1.1

NA. Not available. * nonsusceptible

S6. AMR profiles of *Salmonella* reported by ISPED program during 2016 to 2020

Antibiotic name	*Salmonella* (n=8,498)				
	2016	2017	2018	2019	2020
Ampicillin	76.6	77.7	76.2	76.5	77.7
Amoxicillin/Clavulanic acid	6.7	24.2	10.7	3.3	9.1
Ampicillin/Sulbactam	38.6	44.5	42.3	33.5	41.3
Ceftriaxone	26.9	25.7	22.5	22.4	20.9
Cefepime	14.2	16.2	11.7	14.6	12.5
Imipenem	0	0	0	0	0
Meropenem	0	0	0	0	0
Ciprofloxacin	12.9	12.2	11.5	10.8	9.8
Levofloxacin	4.4	3.8	2.7	5.4	7.5
Trimethoprim/Sulfamethoxazole	25	36.8	37.1	36.1	38.4
Chloramphenicol	32	34.6	32.5	37.2	45
